# Comment on “Intermittent Hypoxic–Hyperoxic Training During Inpatient Rehabilitation Improves Exercise Capacity and Functional Outcome in Patients With Long Covid: Results of a Controlled Clinical Pilot Trial” by Doehner et al.—The Authors' Reply

**DOI:** 10.1002/jcsm.13866

**Published:** 2025-06-13

**Authors:** Wolfram Doehner, Per Otto Schueller

**Affiliations:** ^1^ Berlin Institute of Health Center for Regenerative Therapies, Charité ‐ Universitätsmedizin Berlin Berlin Germany; ^2^ German Heart Center of the Charite, Campus Virchow Klinikum Charité Universitätsmedizin Berlin, German Centre for Cardiovascular Research (DZHK) ‐ Partner site Berlin Berlin Germany; ^3^ Center for Stroke Research Berlin (CSB) Charité Universitätsmedizin Berlin Berlin Germany; ^4^ Klinik für Kardiologie und Pneumologie MEDIAN Klinikum Flechtingen Berlin Germany

To the Editor,

In their letter to the editor, Kulka and Tryba discuss a recent clinical pilot study investigating the efficacy and safety of intermittent hypoxic–hyperoxic training (IHHT) and raise concerns that the nonrandomized group allocation in this study may limit the generalizability of the findings to broader patient populations with long COVID or with myalgic encephalomyelitis/chronic fatigue syndrome (ME/CFS) [1]. In this study, IHHT was applied exclusively to patients with long COVID who had severely impaired functional capacity [2]. The results showed that IHHT, as an additional treatment within a standardized inpatient rehabilitation program, was associated with improvements in functional capacity, health‐related quality of life and both objective and subjective measures of symptom status. IHHT was demonstrated to be safe, well‐tolerated and feasible within the context of an interdisciplinary rehabilitation program. Patients were allocated to the intervention group (receiving IHHT in addition to standard rehabilitation) or to the control group (receiving standard rehabilitation only) based on the clinical judgement of the attending physician. The two groups differed in some baseline characteristics.

Kulka and Tryba argue that these baseline differences suggest that the two groups may not represent the same patient population, potentially limiting the ability to draw conclusions regarding the treatment effect of IHHT. Contrary to this assumption, all patients in this single‐centre study were enrolled by the same study team according to uniform inclusion criteria and following a prospectively defined study protocol. Only patients with a primary diagnosis of long COVID were included. While some baseline differences were observed (e.g., age, 6‐min walk test [6MWT] distance, HDL levels and eGFR), the groups were similar in most clinical variables, including body composition, cardiovascular measures, respiratory capacity, concomitant medications for comorbidities and biochemical parameters. All patients were hospitalized in the same rehabilitation centre during the study and received the same standardized multidisciplinary rehabilitation program, which included physical rehabilitation (breathing exercises, endurance and strength training), relaxation techniques, education, occupational therapy, psychological counselling and optimized management of comorbidities. All patients received the same daily routines and dietary regimen. The only difference in treatment between groups was the addition of IHHT in the intervention group, administered according to a prospective treatment protocol as outlined in the study report. The statistical impact of this intervention was analysed using ANCOVA, assessing changes in functional capacity from baseline and adjusting for baseline measures. Thus, baseline differences were appropriately accounted for. This analysis revealed a significant benefit in the intervention group not only for the 6MWT distance (the primary endpoint) but also across a range of secondary endpoints for which no baseline differences were observed.

Indeed, a battery of functional tests (6MWT, stair‐climbing power), symptom scores (dyspnoea, fatigue, patient global assessment) and quality‐of‐life assessments (EQ‐5D) all demonstrated a beneficial effect of IHHT compared to standard care. Moreover, further exploratory endpoints (maximum handgrip strength, nine‐hole peg test, functional ambulatory capacity and respiratory function) as well as clinical and biochemical variables (e.g., blood pressure, haemoglobin, fasting blood glucose) showed improvements in the IHHT group that were not observed in the control group.

This broad range of symptomatic and functional improvements supports the robustness of the findings and makes it unlikely that the observed benefit in the primary endpoint occurred by chance. However, it cannot be entirely excluded that baseline differences—stemming from the non‐randomized and open‐label study design—may have influenced the observed treatment effect. This limitation is explicitly acknowledged in the study and is the reason why it is considered a pilot study.

In fact, one of the stated aims of this pilot study was to generate real‐world clinical data on the feasibility, safety and efficacy of this innovative treatment approach to support the underlying hypothesis. Notably, this step was explicitly requested by potential sponsors in preparation for a subsequent controlled, blinded, randomized clinical trial. If the benefits of IHHT are confirmed in such studies, broader implementation in other rehabilitation settings and in combination with novel treatment approaches such as SGLT2 inhibitors should be further investigated.

Finally, the expectation expressed by Kulka and Tryba that the findings from this pilot study could be extended to patients with ME/CFS should be approached with great caution. Only patients with long COVID were included in the study—none with ME/CFS. Patients with ME/CFS often experience severe exercise intolerance and post‐exertional malaise (PEM) even after mild physical activity. Whether respiratory stress conditioning—even in short‐term form as applied here via IHHT—can be tolerated by patients with ME/CFS remains unclear and cannot be concluded from this pilot study.

Despite the acknowledged limitations of this investigation, the observed improvements of functional capacity and symptom burden in response to IHHT among patients with long COVID are promising and warrant further evaluation in a controlled, randomized clinical trial.

## Conflicts of Interest

W. Doehner reports consulting fees and speaker honoraria from Bayer, Boehringer Ingelheim, Boston Scientific, Cardiomatics, Aimediq, Medtronic and Vifor Pharma, travel support from Pharmacosmos and research support to the Institute from EU (Horizon 2020), German Ministry of Education and Research, German Centre for Cardiovascular Research, German Pension Insurance (regional Mitteldeutschland), Boehringer Ingelheim and Vifor Pharma. P. Schüller declares consulting fees, speaker honoraria and research support from Pfizer, Amgen, Amarin and Novartis.
